# Fruit development and ripening

**DOI:** 10.1093/jxb/eru307

**Published:** 2014-07-28

**Authors:** Graham B. Seymour, Antonio Granell

**Affiliations:** University of Nottingham, United Kingdom; IBMCP, CSIC-Universidad Politécnica Valencia, Spain

It has been 12 years since publication of the last *Journal of Experimental Botany* Special Issue on Fruit Development and Ripening (Vol. 53, No 377, October 2002). At that time the biosynthesis and mode of action of ethylene in fruit ripening had already been established, and advances in genetics were revealing links between genes and phenotypes, especially noteworthy was the map-based cloning of genes underlying tomato non-ripening loci such as *ripening inhibitor* (*rin*). Since then there have been substantial advances in our understanding of ripening in tomato and many other dry and fleshy fruits. This has been accelerated by the delivery of genome sequences for a wide range of plants including fleshy fruit bearing species and the development of systems biology approaches to understanding regulatory networks.

The first paper in the 2002 issue was a seminal work by Sandy Knapp focused on fruit diversity in the Solanaceae and highlighting the phylogenetic relationships between dry and fleshy fruit forms (Knapp, 2002). The current volume describes the progress that has been made in understanding the mechanistic basis of fruit development and ripening and the conservation of regulatory networks controlling these processes, in both dry and fleshy forms across a wide range of taxa. Systems biology approaches have begun to reveal the complexity of the ripening process, while genome sequences have facilitated the identification of genes underlying quantitative trait loci (QTL) and the importance of epigenetics is beginning to become apparent.

In the first paper in this special issue, Sofia Kourmpetli and Sinéad Drea from the University of Leicester in the UK review the regulatory networks involved in the development and maturation of two, at first sight, very different dry fruits, the poppy capsule and the cereal grain. They highlight the importance of MADS-box genes including *FRUITFULL* (*FUL*) and *SHATTERPROOF* (*SHP*), and set these events in the context of a phylogenetic framework. Cristina Ferrándiz and Chloé Fourquin from Instituto de Biología Molecular y Celular de Plantas in Valencia, Spain, then review the role of FUL and SHP in *Arabidopsis* and provide a comprehensive discussion of their role in many other species including Brassicas, legumes and also in fleshy fruits (Ferrándiz and Fourquin, 2014). Dehiscence in dry fruits and ripening, and even the extent of lignification in fleshy fruits, is linked to the complex relationship between *FUL* and *SHP* expression, and their review concludes that there are conserved roles of FUL and SHP in late fruit development both in fleshy and dry fruits. These ideas were hinted at in the 2002 Special Issue and strong supporting evidence is now presented consistent with dehiscence and ripening sharing a common origin and being parallel, rather than completely different processes. María Dolores Gómez and colleagues, also from Valencia, explore and develop for us further ideas about the similarities and differences between the mechanistic basis of ‘ripening’ and ‘over-ripening’ in dry and fleshy fruits including comparing the transcriptomes of senescent and ripening *Arabidopsis* siliques and tomato berries (Gómez et al., 2014). 

Molecular networks controlling the ripening of fleshy fruits are also the focus of reviews by scientists from a range of European and South American laboratories (Karlova *et al.*, 2014; Kuhn *et al.*, 2014). Karlova and colleagues present the state-of-the-art for the model fleshy fruit, tomato, and bring to our attention recent discoveries relating to the role of the epigenome in controlling the ripening process. Tomato is a climacteric fruit where ripening is under the control of ethylene and a range of transcription factors and other developmental cues. In contrast, Kuhn *et al.* describe the process in the non-climacteric grape berry including an in-depth discussion of the key processes associated with grape maturation such as flavonoid biosynthesis and volatile development. The authors provide an excellent summary of the profound effect of the environment on grape berry ripening, and a review of the role of various hormones in the ripening process in this species where ethylene seems to have an effect even on the ripening of this non-climacteric fruit. The role of hormones other than ethylene in modulating fleshy fruit ripening has received relatively limited attention, although the effects of auxin as a ripening inhibitor are perhaps best known. The reviews by Kumar *et al.* (2014) and Leng and colleagues (Yuan *et al.*, 2014) bring us up to date with current work and indicate that the interplay between hormone signalling and ripening is complex and still poorly understood. The evidence emerging from transgenic and other experiments suggest that ABA is a promoter of ripening in both climacteric and non-climacteric fruit. 

The importance of the plastid in ripening fruits is the subject of the review by Maria Florencia Cocaliadis and her colleagues from Valencia (Cocaliadis *et al.*, 2014). A major question discussed at length is whether chloroplasts in fruits are photosynthetically active to any significant degree and, as a result, whether or not they contribute to carbon accumulation in wild type fruits, or whether this is case dependent as for fruit engineered to accumulate higher numbers of active chloroplasts or mutants deficient or overproducing chloroplast in the fruit. This leads to an interesting discussion on the role of oxidative stress during normal ripening and the fine controls that need to be imposed on the chloroplast to prevent oxidative stress running out of control. Plastids are the sites of synthesis of many important secondary metabolites and volatile compounds, which are the subject of the reviews by Takayuki Tohge *et al.* from the Max-Planck-Institute of Molecular Plant Physiology in Germany and José L. Rambla and colleagues from Valencia and the Netherlands (Tohge *et al.*, 2014; Rambla *et al.*, 2014). In the first review, the links with the wealth of health-promoting secondary metabolites is described, while the second discusses the mechanisms controlling the production of volatile compounds, and approaches to identify and improve the levels of those impacting flavour and aroma.

The final three review papers focus on genes regulating the genetic basis of fruit morphology in horticultural crops and the genetics, form and function of fruit cuticles. Antonio J. Monforte and colleagues from Spain and the USA describe the identification of genes controlling size and shape of tomato using QTL map-based cloning and how this information can be used to understand the basis of these traits in fruits such as melon (Monforte *et al.*, 2014). Laetitia B. B. Martin and Jocelyn K. C. Rose from Cornell University in the USA provide an in-depth review of the form and function of the fruit cuticle (Martin and Rose, 2014) and this is complemented by the review of Shelly Hen-Aviv and colleagues from Israel, Italy and South Africa that discusses the mechanistic basis of cuticle formation and properties (Hen-Avivi *et al.*, 2014). 

The last papers in this special issue describe original research from Dai *et al.* (2014) from France and Fu *et al.* (2014) from China and the UK. Dai *et al.* report on the development of a two-step *in vitro* culture system for grape which couples the use of fruiting-cuttings with organ *in vitro* culture, while Fu and colleagues explore the roles of the phytoene synthase gene family in loquat. The new volume reflects the advances in technology since 2002 and the reader can now survey the discoveries concerning the molecular networks in a range of dry and fleshy fruits. 
